# Smart Hydrogels Meet Carbon Nanomaterials for New Frontiers in Medicine

**DOI:** 10.3390/biomedicines9050570

**Published:** 2021-05-18

**Authors:** Simone Adorinni, Petr Rozhin, Silvia Marchesan

**Affiliations:** 1 Chemical and Pharmaceutical Sciences Department, University of Trieste, 34127 Trieste, Italy; simone.adorinni@phd.units.it (S.A.); petr.rozhin@phd.units.it (P.R.); 2 National Interuniversity Consortium of Materials Science and Technology (INSTM), University of Trieste, 34127 Trieste, Italy

**Keywords:** hydrogels, carbon nanotubes, graphene, carbon nanomaterials, nanostructures, wearable electronics, drug delivery, tissue engineering, regenerative medicine

## Abstract

Carbon nanomaterials include diverse structures and morphologies, such as fullerenes, nano-onions, nanodots, nanodiamonds, nanohorns, nanotubes, and graphene-based materials. They have attracted great interest in medicine for their high innovative potential, owing to their unique electronic and mechanical properties. In this review, we describe the most recent advancements in their inclusion in hydrogels to yield smart systems that can respond to a variety of stimuli. In particular, we focus on graphene and carbon nanotubes, for applications that span from sensing and wearable electronics to drug delivery and tissue engineering.

## 1. Introduction

### 1.1. Hydrogels

Hydrogels are soft materials that retain high levels of water that are widely used for their adsorption and delivery properties in areas such as drug [1] or protein [2] release, tissue engineering [3,4], and wound healing [3,5]. They are typically composed of a three-dimensional network that traditionally arises from chains of polymers [6], polysaccharides [7], proteins [8], or other macromolecules. In recent years, supramolecular hydrogels, whose matrix is based on non-covalent interactions between small molecule building blocks, have attracted increasing attention to attain responsive materials [9] and 3D constructs [10]. One obvious advantage of this latter class is the possibility to easily break and reform the bonds that constitute the hydrogel, so that the material can be dynamic in response to a variety of stimuli [11]. This feature is particularly attractive for advanced applications such as targeted cancer therapy [12], regenerative medicine [13], and wound management [5]. Another advantage is the fine control that is possible to attain over hydrogel chemical constituents, contrarily to, for instance, macromolecular polymers that display a distribution of molecular weights and functional groups’ density [14].

Depending on the origin of the hydrogel components, they can be distinguished in natural and synthetic [15], or semi-synthetic [16]. Among their many properties, injectability, and self-healing are particularly sought after [17], as well as bioadhesion [18,19] and, obviously, biocompatibility [20]. Further, research is very active towards smart hydrogels that can adapt in response to changes in pH [21], temperature [22], light-irradiation [23], chemicals’ [24] or biomolecules’ [25] concentrations, as well as other physico-chemical stimuli [26,27]. A myriad of approaches has been developed to attain these properties, and, amongst them, a popular option consists of multi-component systems with nanofillers [28] to yield nanocomposite [29,30] or hybrid [31,32] hydrogels, so that new properties can emerge from the combination of the different constituents.

### 1.2. Carbon Nanomaterials

**Figure 1:**
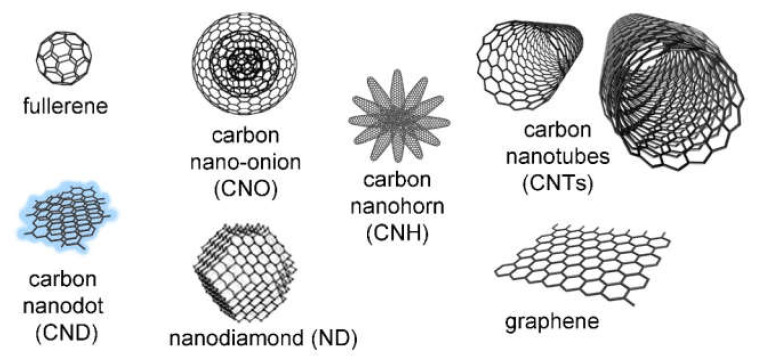
Carbon nanostructures (not to scale), reproduced from Adorinni, S. et. al. (2021) [41] under a Creative Commons license (https://creativecommons.org/licenses/by/4.0/). The nano-onion schematic structure is reproduced with permission from Ugarte, D. et al. (1996) [42], copyright ©1995 Elsevier.

Carbon nanomaterials (Figure 1) are very diverse both in terms of their structure and morphology while being all composed of carbon atoms, which are, in the majority of cases, covalently bound in a sp^2^ hexagonal lattice [33]. A simple approach to understand their structure consists of considering the two-dimensional (2D)-sheet of graphene as a common building block, which can be folded in different ways to form the various carbon materials [34], just like a sheet of paper can be rolled into a nanotube or folded into a ball. In particular, 0D fullerenes [35] can be considered as soccer-ball structures, and 1D carbon nanotubes (CNTs) [36] have a tubular morphology. Other well-studied examples include nano-onions (CNOs) [37] that are formed by concentric fullerenes, and nanohorns (CNHs) [38] that are clusters of nanocones. More recently, carbon dots have attracted great attention for their ultrasmall size (<10 nm) that confers them with peculiar luminescent properties [39]. Nanodiamonds (NDs) differ for they contain a large portion of sp^3^ carbon atoms [40].

All these carbon nanomaterials display specific morphology, size, and reactivity; thus the resulting physico-chemical properties vary greatly from one another. Despite the vast literature on the topic, it is not always straightforward to anticipate which is the most suited for a specific application, especially in the complex context of biologically-relevant samples [43,44,45], or when interacting with biomolecules [46]. Nevertheless, it is possible to state that they all generally feature good electronic conductivity, low density, high mechanical strength, and the ability to be chemically functionalized to tailor their properties as required for the intended use [47]. Their high-surface area and hydrophobic nature can be convenient to non-covalently bind large amounts of bioactive compounds, for instance for drug delivery applications [48]. For all these reasons, they clearly hold a great innovative potential in challenging areas of medicine [49,50], such as the fight against micro-organisms [51] and cancer [52], owing to their targeting ability to reach the tumor microenvironment [53]. They are also very promising for tissue engineering [54], especially for bone [55], and for conductive cells, such as the cardiac [56,57] and nerve tissues [58,59]. Their potential uses in clinical applications [60] and sensing [61] have been recently reviewed. Therefore, this article will focus solely on the very latest developments regarding innovative biomedical uses of carbon nanomaterials in the form of smart hydrogels.

The biomedical application of carbon nanomaterials requires first a good understanding of their interactions with biomolecules, especially proteins [62] forming a corona on the nanocarbon surface [63], and thus affecting the biodistribution [64], the immune response [65], and the biodegradation [66,67] of the nanomaterials. Despite the great advances in all these sectors, concerns remain regarding the nanocarbons’ toxicity [68,69], and one further difficulty for its proper assessment is the great heterogeneity of this class of materials [70]. The lack of unified standards for their production and classification, which is a common issue for nanomaterials [71], is on the agenda of many committees that are working on initiatives to resolve it [72].

## 2. Recent Advancements on Hydrogels with Carbon Nanomaterials for Medicine

The inclusion of carbon nanomaterials in hydrogels is a promising strategy to attain advanced biomaterials for applications in medicine [73]. They are typically used to impart hydrogels with enhanced mechanical properties as well as conductivity, although conductive hydrogels can be attained also using conductive polymers, which are recently gaining momentum in biomedical research [74,75]. Furthermore, carbon nanomaterials’ antimicrobial properties are convenient for various applications in the health sector, ranging from water disinfection [76] to bioactive scaffolds and skin bandages [77]. Also, fabrication strategies, such as nanogel formulations, can maximize the benefits of the properties that arise by working at the nanoscale, especially related to optoelectronic activity and luminescence, for instance for biosensing, bioimaging, and multi-responsive drug delivery systems [78].

**Figure 2:**
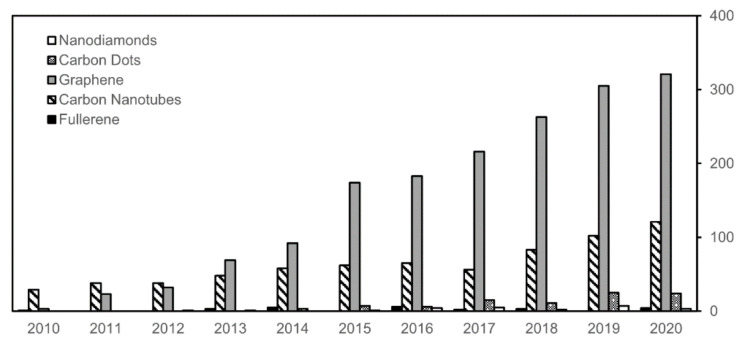
Results of a literature search (22 April 2021) on Scopus for documents over the last decade with the keywords “hydrogels” and one of the carbon nanomaterials as shown in the legend. The results pertaining nano-onions, nanohorns, or nanodiscs were <10 in total and are omitted.

Despite the various types of carbon nanostructures available as described above, the vast majority of research studies on hydrogels with carbon nanomaterials focuses on graphene and, to a lesser extent, on carbon nanotubes (Figure 2). Therefore, this review will focus on these two types of carbon nanostructures. It is apparent that even though CNTs were discovered before graphene, they are lagging in terms of number of studies for this kind of applications. This is likely the result of the concerns raised over their morphological similarity to asbestos fibers, even though it is demonstrated that chemical functionalization [79] and fine control over the hydrogel stiffness [80] can alleviate their pathogenicity. Furthermore, given the large diversity of available CNT types [70], and the fact that their biocompatibility depends on a plethora of factors [81], it becomes apparent that alarming generalizations that pose innovation barriers should be avoided [82].

### 2.1. Hydrogels with Graphene-Based Materials

Graphene-based materials come in different forms and their classification is discussed elsewhere [83]. Graphene’s unique electronic and mechanical properties have stimulated scientists’ imagination for a myriad of biomedical applications, although unsolved challenges remain for its large-scale production on a global scale for implementation in the biomedical sector [84]. Graphene can display an exceptional Young modulus of 1 TPa, remarkable surface area as high as 2630 m^2^/g, and an extraordinary electrical conductivity of 6000 S/cm [85]. It is thus not surprising that numerous studies have focussed on the applications of such properties to open new horizons in the biomedical field as summarized in Table 1. Below, the most recent advances are briefly described divided by type of envisaged application.

#### 2.1.1. Wearable Electronics and Artificial Skin

**Figure 3:**
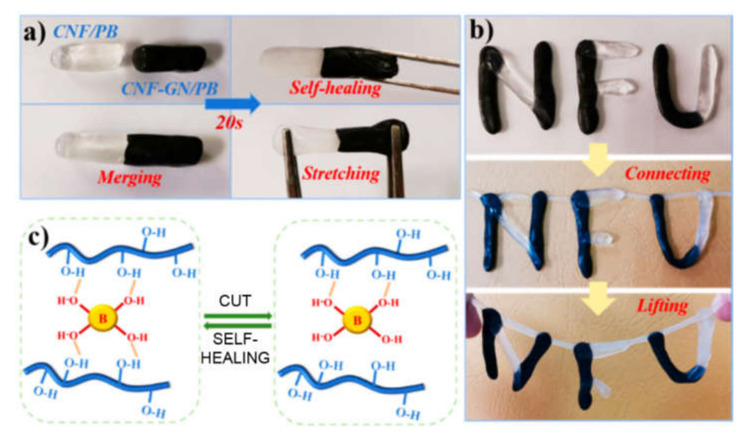
Self-healing GO-PVA hydrogel. (**a**,**b**) examples of the self-healing ability. (**c**) self-healing mechanism based on the use of borax as cross-linker between hydroxyl groups of PVA polymer. Reproduced from Zheng, C. et al. (2019) [87], under a Creative Commons license (https://creativecommons.org/licenses/by/4.0/).

One of the most innovative uses of graphene’s properties focusses on the development of wearable electronics, given its high conductivity, stability, low density, and flexibility [103]. With the advent of smart phones and watches, the technology is getting ever closer to the human body, and it is moving in the direction of being incorporated into smart textiles, or even in skin patches and towards the generation of artificial skin as a replacement for irreversibly damaged tissue. Materials designed for this kind of purpose need to satisfy numerous demands in terms of mechanical properties, electrical performance, and biocompatibility. Therefore, new approaches are continuously sought to push the leading edge of research a step further. For instance, it was recently found that the inclusion of calcium hydroxide nanoparticles into a polyacrylamide-reduced graphene oxide (rGO) hydrogel led to the development of a strain sensor with good stretchability as required for wearable electronics [100]. The smart material responded to mechanical deformations by displaying changes in resistivity, which allowed the use as strain sensor [100]. Calcium ions also proved useful for the performance of a polyvinyl alcohol (PVA)-based hydrogel, where they served as cross-linkers; the material, with silver nanowires and graphene oxide (GO), was developed to create an artificial skin, with the ability to sense pressure variations [92]. Alternatively, borax was used as crosslinker for PVA which, combined with cellulose nanofibers and graphene, yielded a strain-sensing material with excellent self-healing ability (Figure 3), which was also envisaged for creating artificial skin [87]. Addition of glycerol provided a PVA-based hydrogel with anti-freezing properties, while a sandwich structure, with the polymer being sandwiched between graphene layers, notably improved the sensitivity of the strain-sensor envisaged for wearable electronics [88].

#### 2.1.2. Nerve and Cardiac Tissue Regeneration

**Figure 4:**
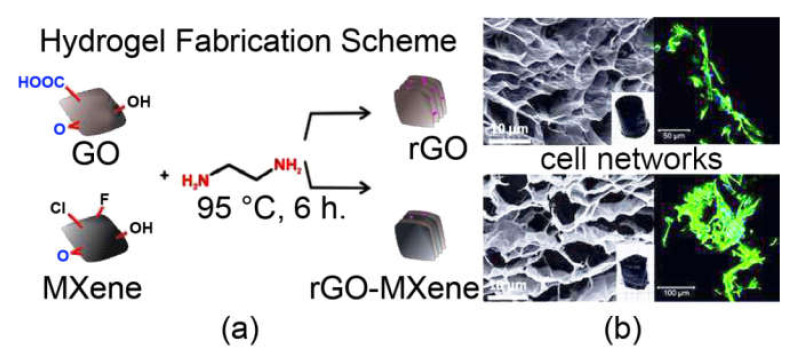
rGO-MXene hydrogels preparation (**a**) as scaffolds for cell networks (**b**). Adapted from Wychowaniec, J.K. et al. (2020) [102], under a Creative Commons license (https://creativecommons.org/licenses/by/4.0/).

Graphene’s high conductivity, low density, and chemical stability, make it a highly researched component also for conductive-tissue regeneration [104,105], such as the nerve [59] and cardiac tissues [106]. To this end, natural biopolymers such as alginate [101] and biodegradable alternatives such as polylactic acid [44], are often preferred, although new synthetic materials are also being investigated. In particular, the whole class of 2D materials is a hot topic of research to advance the frontiers of tissue replacement, thanks to their low density and high mechanical resistance [107]. As an example, a titanium carbide (belonging to the family of 2D-materials called MXenes) was used to yield a hydrogel with rGO flakes (Figure 4) that demonstrated an excellent ability to sustain cell culture for the envisaged future application of heart actuators for cardiac repair [102]. Other approaches rely on multi-layered and multi-component structures to provide hydrogels with different spatio-temporally resolved responses, as needed to perform the complex task of tissue regeneration. For example, recent advancements included the design of a core-shell GO microfibrillar hydrogel with a chemoattractant, a growth factor, and nucleic acids, to perform the gene-transfection of endogenous stem cells that were effectively recruited and differentiated to reconstruct cutaneous nerves [94]. The hydrogel was 3D-printed as microfiber arrays, which were then crosslinked into a hydrogel chip. The smart material responded to recruited cells, since it displayed chemical bonds that were hydrolyzed by proteases secreted by the stem cells; as a result, the material released the gene vector and growth factor that induced differentiation into a neural-like lineage [94].

#### 2.1.3. Protein Adsorption/Crosslinking for Bone/Muscle Repair or to Prepare Specialized Food Products

Another useful property of graphene is its high surface area, which can be conveniently exploited for the adsorption of biomolecules. To this end, a GO-loaded poly(*N*-isopropylacrylamide)-chitosan hydrogel was enriched with growth factors and, taking advantage of the thermo-responsive behavior of the synthetic polymer, demonstrated a good performance towards in-situ angiogenesis for regenerative medicine [91]. The gel precursor solution could be loaded with cells and growth factors into a syringe, so that, upon injection in vivo, the smart polymer responded to the body temperature by undergoing gelation into a scaffold to sustain neovascularization [91]. Similarly, the ability of GO to adsorb proteins was used to engineer a fibrin hydrogel for bone regeneration, which was loaded with hydroxyapatite to favor biomineralization, and with iron-oxide nanoparticles to render the system magneto-responsive [96]. Alternatively, bioactive proteins can be covalently bound to the large surface provided by graphene and stabilized through encapsulation within a hydrogel matrix. For example, galactosidase was crosslinked to GO flakes and formulated as alginate gel to prepare lactose-free food products [95].

As mentioned above, poly(*N*-isopropylacrylamide) is a convenient polymer that has gained a lot of attention for biomedical use to achieve smart hydrogels with graphene that respond to heating to human-body temperature through a physical collapse of the polymer chains, thus releasing entrapped drugs [108]. A laser-engraving system can be used to render these materials conductive through a so-called laser-induced graphitization [109]. Microfluidics proved to be effective for producing a hydrogel with GO, poly(*N*-isopropylacrylamide), and alginate to yield a thermo- and electro-responsive material with reversible bending properties for potential applications in the development of artificial muscles [110]. The synthetic polymer caused volume changes in response to temperature variations, while the natural polymer determined bending in response to electrical stimulation. This phenomenon occurs as a result of the fact that the polyelectrolyte macromolecules remain immobile, while their counterions move towards their counter electrodes. The consequent ionic concentration gradient that arises in the direction of the electric field determines a difference in osmotic pressure within the hydrogel that provides the driving force for bending [110].

#### 2.1.4. Cartilage and Ligament Regeneration

Growth factors can be adsorbed onto the large surface area of graphene or GO in high amounts to direct stem cell differentiation. This approach was demonstrated for hydrogels based on GO and either collagen [111] or poly-D,L-lactic acid/polyethylene glycol [112], designed to repair the cartilage. Chondroitin sulfate in another biomolecule of interest to promote regeneration of this tissue, and to this end it was crosslinked with a chemically modified GO, to yield a hydrogel that effectively stimulated the deposition of a collagen matrix from mesenchymal stem cells [113]. Hydroxyapatite is another useful component of natural origin to yield biomaterials to repair the cartilage, as shown for a hydrogel with GO and polyvinyl alcohol that was 3D printed and displayed excellent biomechanical and bio-friction properties [114]. Indeed, the presence of GO can be beneficial to increase the fidelity and resolution of 3D printed scaffolds [115]. Further, GO can increase the lubrication properties of the biomaterial, as demonstrated on a multilayered system based on gellan gum and poly (ethylene glycol) diacrylate hydrogel [116]. Alternatively, modern plasma techniques can be used to generate radicals and crosslink hydrogels for cartilage reconstructive surgery, as applied to a gelatin-GO gel [117]. 

#### 2.1.5. Eye Regeneration and Mimicry

Thermo-responsive polymers are widely applied to regenerate the eye and to create artificial bio-actuators for biomimicry. To this end, a hyperelastic poly(*N*-isopropylacrylamide) hydrogel was formulated with GO and shaped as an iris, with a hole in the middle. This actuator was designed to mimic the human iris’ action in response to light. Upon illumination, the photo-thermal conversion effect of GO led to a decrease in size of the inner hole, with a consequent decrease in transmitted light as the incident light intensity increased [118]. Methacrylate hydrogels have also been proposed as components to attain lenses, and polyvinylpyrrolidone and GO nanoparticles demonstrated high wettability as additives, allowing also for an ultraviolet shielding effect [119].

#### 2.1.6. Drug Release

Hydrogels made of GO and poly(*N*-isopropylacrylamide) display also photo-responsiveness, because they can deform in response to infrared-light irradiation, thanks to GO’s presence [120]. The two components can be crosslinked for improved mechanical properties [121], and to achieve controlled drug release thanks to the dual responsiveness to both pH changes and electrical impulses [122]. Poly(*N*-isopropylacrylamide-co-methylacrylic acid copolymer-derived GO hydrogel was also successfully used to attain thermo- and pH-responsive membranes for controlled drug release [123]. Alternatively, reduced GO (rGO) can be used to enhance the material conductivity [124,125]. 

Electro-responsive properties have been envisaged also for skin bandages, so that drug release can be controlled upon application of a small voltage [89]. To this end, hydrogel films were prepared using acrylamide, polyethylene glycol, GO, gelatin or trypsin, and curcumin to attain antibacterial activity against methicillin-resistant *Staphylococcus aureus* [89]. Interestingly, when low voltage (12–24 V) was applied, the gels swelled as a result of the ionization of the proteins’ acidic groups above their isoelectric point, while when higher voltage (48 V) was used, the system shrunk because of the predominant effect determined by the osmotic pressure generated by mobile ions movement across the gel network [89]. 

**Figure 5:**
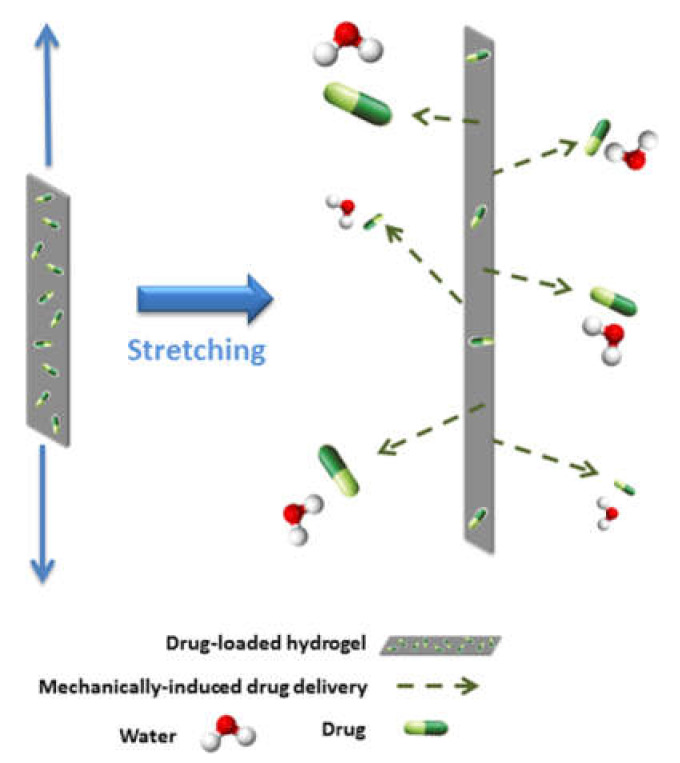
Example of mechanically responsive graphene hydrogel for drug release. Adapted with permission from Gonzalez-Dominguez, J.M. et al. (2018) [86] Copyright © 2021, American Chemical Society.

Besides electro-responsiveness, mechanical responsiveness can also be exploited to attain drug release upon stretching of a hydrogel (Figure 5), as shown for a hybrid material composed of graphene and polyacrylamide [86]. Other convenient stimuli can be changes in pH or ionic strength, as demonstrated for a hydrogel made of polyacrylamide, carboxymethylcellulose, and GO [90]. Similarly, pH-responsive drug release was shown for the anti-cancer 5-fluorouracil loaded in a GO-pluronic hydrogel where the polymer was derivatized with oligolysines to bind GO through electrostatic interactions [126]. pH-sensitive drug release was shown also for a cellulose hydrogelator loaded with GO and prepared through a Pickering emulsion approach [127].

A field where targeted drug release is particularly important is obviously cancer therapy. Among the various hydrogelators, chitosan is widely used in virtue of its low cost, biocompatibility, and ease of derivatization thanks to the presence of reactive amine groups [128]. Upon suitable functionalization and crosslinking, smart hydrogels can be attained from this natural biopolymer [129]. Combined with polyethylene glycol and rGO, it yielded a photo-responsive hydrogel that was envisaged for cancer therapy, as it could be loaded with doxorubicin, which was released through various stimuli, including changes of pH. In particular, at the acidic pH of 6.5 as found in cancer cells, the drug release proceeded more efficiently relative to the use of physiological pH 7.4 as found in healthy tissues [99].

**Figure 6:**
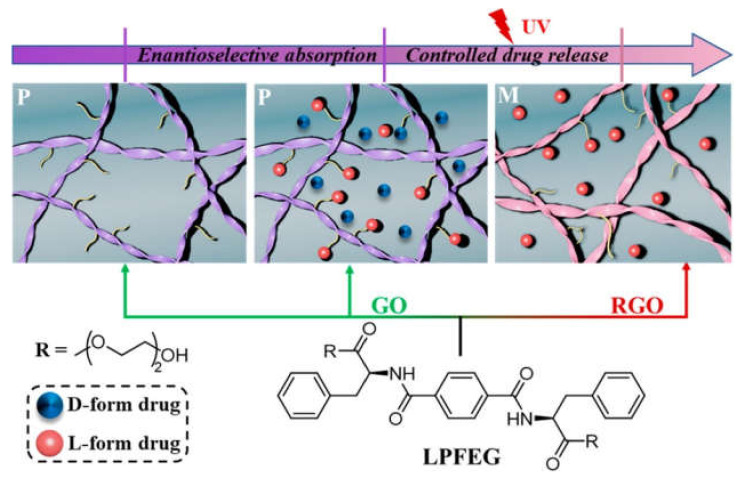
Photo-triggered drug release from a phenylalanine derived hydrogelator that self-assembles into helical nanoribbons on the surface of GO flakes and allows for enantioselective drug adsorption. Reprinted with permission from Zhang, Y. et al. (2020) [98], Copyright © 2021 American Chemical Society.

Self-assembling peptide hydrogels are also interesting candidates to attain drug release [130]. They have been combined with graphene-based materials for improved mechanical properties. For instance, a self-assembling tripeptide sequence, which proved useful as a vehicle for anticancer [131], anti-inflammatory [132], or antibiotic [133] drugs, displayed enhanced stiffness and mechanical resistance upon interaction with GO [46]. The mechanical resilience of multi-responsive bio-elastomers based on resilin polypeptides was also attained with GO [134]. Using a pseudopeptide gelator combined with graphene further yielded a thermo-responsive system [135]. These gelators are attractive not only for their molecular simplicity and ease of preparation, but also for their chirality-induced effects. For instance, a phenylalanine-derivative hydrogelator was shown to self-assemble into right-handed helical nanoribbons on the surface of GO flakes [98]. UV-irradiation determined the switching of helicity to left-handed, and this phenomenon was applied for the enantioselective adsorption and subsequent smart release of chiral drugs (Figure 6), such as ibuprofen, upon photo-stimulation [98].

#### 2.1.7. Electrode Sensors for Disease Monitoring

Carbon-based materials are ideal components to build flexible sensors for instance to monitor physiological parameters [136]. The amino acid cysteine was employed to yield a graphene-hydrogel electrode that was envisaged to monitor pathological states such as diabetes and obesity [97]. The presence of a glucose transporter on cells’ surface could be detected upon recognition by a specific antibody coupled to carbon dots, thus leading to electrochemiluminescence [97]. Therefore, the system exploited both the electro-responsiveness of graphene for the electrode generation, and the photo-responsiveness of carbon dots for the visual detection.

### 2.2. Hydrogels with Carbon Nanotubes (CNTs)

The anisotropic structure of CNTs can offer additional advantages relative to graphene, as shown for instance in the superior performance of CNT-derived biomaterials to reconnect neurons, whose bioelectric activity is boosted when grown on CNT scaffolds [58]. CNTs’ elongated structure favors the alignment of electroactive cells, such as cardiomyocytes, which in turn increases their contractility along the long axis as in the cardiac tissue, thus offering good biomimicry for heart repair [57].

CNTs are popular additives to reinforce composite matrices [137] and can be spun into fibers for uses as conductive wires for wearable electronics [138] or implantable biosensors to monitor neural activity [139]. Furthermore, CNT fibers can easily be functionalized in a myriad of ways [140], including in the convenient waste-free gas-phase [141]. Many applications can be envisaged, especially when CNTs are embedded in smart hydrogels, and recent advances in this area are summarized in Table 2. It is apparent that the majority of studies focus on multi-walled CNTs, which consist of multiple sheets of graphene rolled up to form concentric nanotubes, and which are the easiest to handle and disperse in aqueous environments.

#### 2.2.1. Wearable Electronics and Artificial Skin

**Figure 7:**
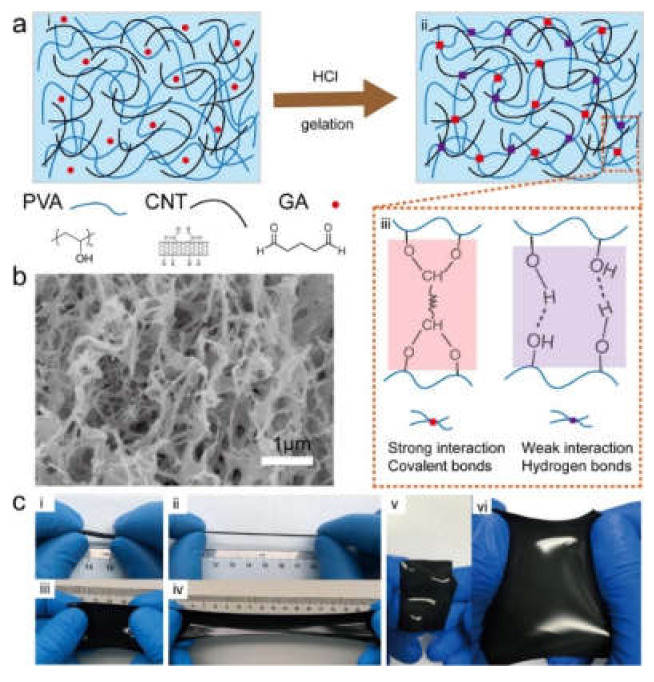
(**a**) Schematic hydrogelation process. (**b**) SEM image of the hydrogel. (**c**) Stretching of the elastic material. Reproduced with permission from Wang, H. et al. (2020) [149] © 2021 Elsevier.

As discussed above for graphene, CNTs find applications in the development of wearable electronics and artificial skin, often through similar approaches. A popular polymer for this kind of applications is PVA, alone or in combination with other polymers. Self-healing properties can be attained by introducing suitable dynamic cross-linkers, so that, through a combination of covalent and non-covalent interactions, the material can achieve a good balance between toughness and ability to self-heal. This was demonstrated for PVA combined with a poly(*N,N*-dimethyl acrylamide) copolymer derivative modified with pyrene and borate functional groups with well-dispersed CNTs and self-healing ability [159]. In particular, the pyrene moiety allowed for π-π interactions between the polymer and the CNTs, while dynamic boronate ester bonds crosslinked the two polymers and enabled self-healing [159]. Alternatively, glutaraldehyde proved to be an effective cross-linker for PVA and CNTs thus yielding a tough yet highly elastic and conductive hydrogel (Figure 7) whose applicability was demonstrated in wearable devices to detect finger motion, to monitor the pulse, and to record electromyograms [149]. Also, calcium divalent cations are effective cross-linkers, as shown on a PVA-alginate-CNT hydrogel whose piezoresistive and piezocapacitive performance allowed sensitive responses to subtle pressure changes in the human body, such as finger or knee flexion, and respiration, and was thus envisaged as integrated strain sensor for skin-like wearable electronics [161].

Fibrous components are very attractive for wearable electronics as mentioned above. Addition of nanocellulose fibers was reported as a useful strategy to improve the mechanical properties of CNT-polymer hydrogel strain sensors for potential applications in wearable electronics and artificial skin development [144,162]. Wet-spinning was used to prepare conductive microfibers from crosslinking of hyaluronic acid with CNTs, with good mechanical properties, electroactivity, and biocompatibility as revealed through implantation in vivo [156].

**Figure 8:**
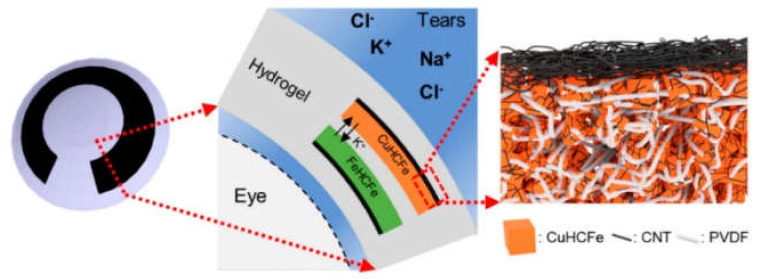
Aqueous battery for smart contact lenses based on polyvinylidene fluoride polymer, CNTs, and Prussian blue analogue nanoparticles CuHCFe and FeHCFe as cathode and anode, respectively. Reproduced from Yun, J. (2021) [160] © 2021 American Chemical Society.

CNTs were recently envisaged also for the fabrication of aqueous batteries for smart contact lenses that operate in tears (Figure 8). To this end, a multilayered structure was necessary. In particular, nanocomposite flexible electrodes were formed by using CNTs and Prussian blue derived nanoparticles to obtain separate sections that acted as a cathode and an anode. The electrodes were encapsulated within a UV-polymerized hydrogel, which acted also as an ion-permeable separator. The power supply was sufficient to operate a low-power static random-access memory, and demonstrated good mechanical stability, biocompatibility, and compatibility with a contact lens cleaning solution [160].

#### 2.2.2. Nerve and Cardiac Tissue Regeneration

CNTs are often added to hydrogels to improve the mechanical properties and impart conductivity, as shown for pH-responsive polymer microparticles that were envisaged for soft tissue repair [143]. Host-guest chemistry enabled the crosslinking of a hydrogel through polyethylene glycol encapsulation within cyclodextrins. Upon irradiation, the hydrogel, which also contained photo-responsive porphyrin and CNTs, disassembled in vivo and was thus proposed as a smart biomaterial scaffold that could respond to light as a stimulus [146].

Once responsive scaffolds are prepared, the next challenge to face is the integration with biological tissue. A popular approach consists of seeding cells prior to implantation, and to this end suitable protocols for the manipulation of hybrid systems with cells and scaffold must be developed. A recent advancement in this direction was provided by the combination of cell-laden methacrylated collagen, alginate matrix, and CNTs for cardiac patches [150]. Importantly, the derivatized collagen could be micro-patterned using a UV-crosslinking protocol in the presence of cells, whilst preserving their viability [150]. CNTs’ presence is important to ensure the scaffold is conductive to lead in-grown cardiomyocytes towards synchronous beating and avoid arrhythmias. This was the case for a recent CNT-hydrogel ingeniously made from pericardial matrix, which allowed the maturation of human-induced pluripotent stem cell-derived cardiomyocytes. The cells displayed enhanced alignment, contraction amplitude, and mature phenotype, with better response to electrical and pharmaceutical stimulation relative to controls [151].

**Figure 9:**
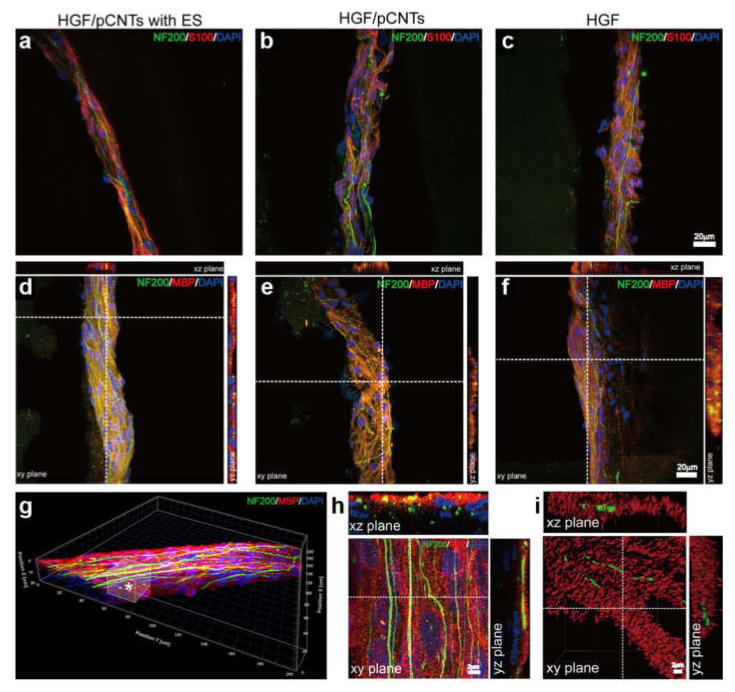
Neuronal axon myelination after 30-day cultivation within peptide-CNT hydrogels. (**a**–**c**) Immunostaining with anti-S100/anti-NF200 antibodies to probe the interactions between Schwann cells and axons and (**d**–**f**) with anti-MBP/anti-NF200 antibodies to probe myelination. (**g**) 3D structure of bundled axons within the hydrogel under electrical stimulation. The asterisk-marked region in (**g**) was investigated by analyzing (**h-i**) higher-magnification sections with axons and myelinated segments colored green and red, respectively. Adapted with permission from He, L. (2020) [153], copyright © 2021, American Chemical Society.

Self-assembling peptides are gathering increasing attention as hydrogel building blocks for their biocompatibility and ability to convey biological messages to cells [163]. Inclusion of CNTs can impart them with self-healing ability, thanks to non-covalent interactions between the amphiphilic gelator and CNTs [46], which in turn favor CNT dispersibility in water [164]. This kind of soft materials has been applied for the regeneration of peripheral nerves and myelination, with good results especially when combined with electrical stimulation (Figure 9) [153].

#### 2.2.3. Bone/Muscle Repair

CNT dispersibility in water could be promoted through adsorption of dopamine on their surface, so that a conductive hydrogel could be obtained with polyethylene glycol diacrylate. The material displayed self-rolling in response to irradiation or humidity. When bone-marrow derived stem cells were cultured on the scaffold, they displayed higher levels of osteogenic differentiation in response to higher levels of CNTs [145].

A strategy that is gaining momentum to control the structure of biomaterial scaffolds from the micro- to the macro-scale is 3D-printing. This fabrication technique was applied on a hydrogel obtained by crosslinking a negatively charged monomer (i.e., p-styrenesulfonate) and a positively charged monomer (i.e., 3-(methacryloylamino)propyl-trimethylammonium chloride) with 2-oxoglutaric acid [165]. After CNT addition to the precursor solution, the resulting polyionic gel was 3D-printed in various of micro-patterns for the culture of bone marrow-derived mesenchymal stem cells. The formation of mineralized matrix and the upregulation of osteogenesis-related genes confirmed a higher degree of osteogenic differentiation for cells grown in the presence of CNTs. Following experiments in a calvarial defect model of rats confirmed that the scaffolds promoted healing relative to controls, thus showing promise for biomedical use in bone tissue repair [166].

#### 2.2.4. Cartilage and Ligament Regeneration and Mimicry

CNTs have been widely studied as useful additives to yield hydrogel scaffolds to repair the cartilage [167]. Besides tissue regeneration, CNTs have been applied on soft robots that mimic muscle and cartilage, through alignment and micropatterning [168]. Indeed, the anisotropic structure of CNTs can be beneficial to attain correct hierarchical organization to recapitulate natural tissues, which is an important aspect also for tendon regeneration [169]. The conductivity of CNTs can be exploited to this end also in the preparation of the hydrogel scaffold, for instance by using electrophoresis to align the CNTs within the biomaterial matrix [170]. 

#### 2.2.5. Eye Regeneration

CNTs are also promising components for inclusion in retinal prosthetic devices. To this end, CNT electrodes successfully stimulated retinal ganglion cells (RGCs) in a mouse model of outer retinal degeneration. Electrophysiological recordings showed a progressive increase of coupling between cells and electrodes over days, thus providing evidence for the formation of viable bio-hybrids between CNTs and the retina [171]. CNTs were also successfully applied as delivery systems for non-viral neurotrophic factor gene therapy to treat glaucoma [172].

#### 2.2.6. Drug Release

Acrylamide-based polymers are widely used to yield pH- and thermo-responsive matrices, as described in the previous sections. For instance, the different ionization state of acidic groups of acrylic acid at different pH values is convenient to attain materials that shrink as the pH is reduced, thus release their embedded components. Coupling an acrylamide-co-acrylic acid polymer with CNTs and chitosan as natural antimicrobial agent indeed yielded pH-responsive hydrogels with antibacterial activity [142].

Hydrogels of natural origin are also widely used. For instance, Matrigel^®^ provided a scaffold for CNTs conjugated to mesoporous silica for the pulsatile drug delivery of an anticancer agent [146]. In particular, doxorubicin was released through a photo-thermal effect that was triggered by near-infrared light irradiation of the responsive CNTs [154]. Whey proteins were recently reported to facilitate CNT dispersion within their hydrogels, with CNTs leading to shortening of the protein fibrils that may have useful implications in the development of innovative therapies for amyloidoses [173].

#### 2.2.7. Electrode Sensors for Disease Monitoring

This research area is widely studied, especially in light of the increasing numbers in terms of ageing population, which often requires monitoring of multiple chronic illnesses to ensure good health. An alanine-based amphiphile hydrogelator was used with redox-active viologen to obtain a hydrogel, and addition of CNTs rendered the system quasi redox reversible for applications in bioelectronics [155]. CNTs were also used to impart photo-responsiveness to a hydrogel that already displayed gel-to-sol transition upon ultrasonication, thus providing a multi-responsive system envisaged for bioelectronics [158]. A poly(N-isopropylacrylamide) thermo- and pH-responsive hydrogel embedding CNTs provided an artificial muscle which, upon inclusion of the glucose oxidase enzyme in the system, displayed responsiveness to glucose as a model biomolecule [147]. Glucose sensing for diabetes patients was also obtained by including glucose oxidase with CNTs in a hydrogel obtained by crosslinking mucin with the enzyme and albumin [152].

**Figure 10:**
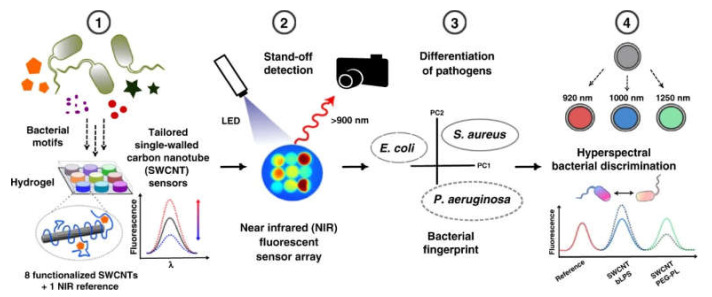
(1) Nanosensors based on near-infrared fluorescent CNTs. (2) Array of eight hydrogel nanosensors and one reference. (3) Bacteria growth changes the sensor array fingerprint, which allows us to differentiate important pathogens. (4) Multiple sensors can be spectrally encoded and used for hyperspectral differentiation of bacteria. Reproduced from Nißler, R. et al. (2020) [157].

Pathogens’ detection is another urgent need that could benefit from CNTs’ inclusion in smart hydrogels. Recently, multiplexed nanosensors were developed by embedding suitably functionalized CNTs within a hydrogel matrix of polyethylene glycol and using the near-infrared fluorescence of CNTs as a means of detection (Figure 10). The CNTs were ingeniously modified with different biomolecules for pathogens’ recognition, so that their presence triggered fluorescence changes upon photoexcitation of the CNTs, thus allowing to detect and identify different types of bacteria [157]. 

## 3. Conclusions

This concise review discussed the most recent examples of the latest progress in the research pertaining graphene’s and CNTs’ inclusion in smart hydrogels to innovate in the health sector. The two materials share common properties and thus find similar types of applications, although their differing nanomorphology and consequent physicochemical differences should be taken into account when designing new materials. In particular, CNTs’ elongated structure appears to provide an additional benefit for the biomimicry of conductive tissues, and for the fabrication of fibrous structures, such as yarns and ropes, and conductive wires.

Among the various types of chemistries used, it is apparent that the combination of covalent and non-covalent chemistries can be a strategic choice to provide tough and durable materials (through covalent bonds) that can also be dynamic in nature, stretch, and self-repair (thanks to non-covalent linkages). Clearly, the most innovative research studies benefit form multi-disciplinary approaches, which can synergize from skills in chemistry and materials science to design smart hydrogels, as well as those in engineering and electronics specially to include a bio-electronic component. Finally, a good knowledge of the biological target of interest ensures appropriate assessment of biocompatibility and of the materials’ effects on cells in a physiological and pathological context. In particular, biosensors that can be integrated in medical implants and devices have significantly advanced and offer the potential to address unsolved challenges in tissue regeneration and biomedical monitoring of biological parameters.

It is worth noting that there are many other carbon nanomorphologies available for research (Figure 1) and that may hold undisclosed potential to innovate in medicine. Therefore, although CNTs and graphene are obvious key players in the development of smart hydrogels, it is certainly worth considering other structures that could provide morphology-related advantages that are yet to be discovered. In conclusion, it is apparent that carbon-based nanomaterials’ inclusion in smart hydrogels led to great leaps forward in terms of technological progress, but their innovative potential still holds unexplored areas that could open new horizons for the benefit of society.

## Figures and Tables

**Table 1:** Recent examples of smart hydrogels with graphene for biomedical applications. GO = graphene oxide, rGO = reduced GO.

Graphene Type	Gelator and Additives	Responsiveness	Application	Ref.
Graphene	Polyacrylamide	Drug release upon stretching	Wound healing	[86]
Graphene	Polyvinyl alcohol-borax, nanocellulose	Strain sensor	Artificial skin	[87]
Graphene	Polyvinyl alcohol, polyacrylic acid, glycerol	Electro-active	Wearable electronics	[88]
GO	Polyacrylamide, polyethylene glycol dimethacrylate	Electro-responsive drug release	Skin bandage	[89]
GO	Polyacrylamide, polyacrylic acid, carboxymethylcellulose	Salts/pH (de)swelling	Drug release	[90]
GO	Poly(*N*-isopropylacrylamide), chitosan, growth factors	Thermo-sensitive	Angiogenesis for tissue regeneration	[91]
GO	Polyvinyl alcohol, Ca^++^ ions, silver nanowires	Pressure sensor	Artificial skin	[92]
GO	Polyvinyl alcohol, G-quartet/hemin	Electro-active	Biosensors	[93]
GO	Cross-linked polyethylenimine	Enzyme-responsive check	Tissue regeneration	[94]
GO	Alginate, galactosidase	Lactose	Lactose-free Foodstuff	[95]
GO	Fibrin, iron-oxide nanoparticles, Hydroxyapatite	Magneto-responsive	Bone regeneration	[96]
GO	Cysteine	Electrochemi- Luminescence	Electrode sensor for diabetes	[97]
GO	Phenylalanine-derivative	Photo-active	Drug delivery	[98]
rGO	Carboxymethylchitosan, polyethylene glycol	Photo-thermal drug release	Cancer therapy	[99]
rGO	Polyacrylamide, calcium hydroxide nanoparticles	Strain sensor	Wearable electronics	[100]
rGO	Alginate	Electro-active	Heart repair	[101]
rGO	MXene (metal carbide)	Electro-active	Artificial heart actuators	[102]

**Table 2:** Recent examples of smart hydrogels with CNTs for biomedical applications. MW = multi-walled. SW = single-walled.

CNT Type	Gelator and Additives	Responsiveness	Application	Ref.
MW	Polyacrylamide-co-acrylic acid, Chitosan	pH	Biomaterial	[142]
MW	Poly(ethyl acrylate-co-methacrylic acid-co-1,4-butanediol diacrylate)	pH	Soft tissue repair	[143]
MW	Polyacrylic acid, nanocellulose	Self-healing	Biosensors	[144]
MW	Polyethylene glycol diacrylate, Dopamine	Photo-triggered self-rolling	Bone regeneration	[145]
MW	Polyethylene glycol, α-cyclodextrin, Porphyrin	Photo-triggered Disassembly	Tissue engineering	[146]
MW	Poly(*N*-isopropylacrylamide), glucose oxidase	Glucose	Biosensors	[147]
MW	Poly(*N*-isopropylacrylamide), polyacrylic acid	Thermo-responsive	Biomaterials	[148]
MW	Polyvinyl alcohol, glutaraldehyde	Self-healing	Biosensors	[149]
MW	Methacrylated collagen, alginate	Electro-active	Cardiac patch	[150]
MW	Pericardial matrix	Electro-active	Cardiac patch	[151]
MW	Mucin, albumin, glutaraldehyde, glucose oxidase	Glucose	Biosensors	[152]
MW	Self-assembling tripeptide	Self-healing	Biomaterial	[46]
MW	Self-assembling 16-mer peptide	Electro-active	Nerve repair	[153]
MW	Matrigel, mesoporous silica, isobutyramide, doxorubicin, albumin	Photo-active	Drug release	[154]
SW	Alanine-based amphiphile, Viologen	Electro-active	Bioelectronics	[155]
SW	Hyaluronic acid, cross-linkers	Electro-active	Bioelectronics	[156]
SW	Polyethylene glycol, biomolecules	Photo-active	Biosensors	[157]
SW	Poly(ethylene glycol)-block-poly(D,L-allylglycine)	Photo-active	Bioelectronics	[158]
SW	Poly(vinyl alcohol), poly(*N,N*-dimethyl acrylamide) copolymer derivative with pyrene and borate groups	Self-healing	Biomaterials	[159]
SW	Polyvinylidene fluoride, CuHCFe, FeHCFe	Electro-active	Contact-lens battery	[160]

## Data Availability

Not applicable.
